# Obituary

**Published:** 2008

**Authors:** P. S. Iyer

**Affiliations:** 8/553, Shreyas, 11^th^ Road East, Chembur, Mumbai 400071, India


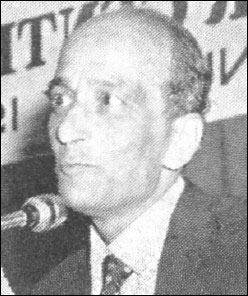


**Dr. K. Sundaram** (1926–2008)

Dr. K. Sundaram, MD, FNA, FASC, Ex-Director, Biomedical Group, Bhabha Atomic Research Centre (BARC) and the first President of the Association of Medical Physicists of India (AMPI), passed away in Coimbatore, Tamil Nadu, India, on May 20, 2008, following a brief illness due to chronic obstructive pulmonary disease.

Dr. Sundaram was born on 28^th^ October, 1926 in Kollengode, Kerala. He had his schooling in SIES School at Sion and later joined Elphinstone College, Mumbai. Dr. Sundaram obtained his MBBS degree in 1949 and MD (Obstetrics and Gynecology) from G. S. Medical College (KEM Hospital, Parel, Mumbai) in 1953. He was the recipient of the Prince of Wales Gold Medal and a fellowship for securing the first rank in the MD examination. Dr. Sundaram joined BARC as Scientific Officer in 1957, was later appointed as Head of the Medical Division in BARC, and retired as Director of the Bio-Medical Group in BARC in November, 1986. During the period of 1979–1982, he was Director of the Division of Life Sciences in International Atomic Energy (IAEA) in Vienna, Austria. His fields of interest included radiation biology, cytology, genetics, cancer, immunobiology, nuclear medicine and effects of high background radiation levels (as in some parts of Kerala), food irradiation, genetics, toxicology, and environmental mutagenesis.

Dr. Sundaram was the recipient of (i) Homi Bhabha award in nuclear medicine, and (ii) Vyas Memorial Oration award in basic medical research. He was a Fellow of the Indian National Science Academy and Indian Academy of Sciences. In addition, he was (i) founder-President of the Society of Nuclear Medicine (1966–1970), (ii) Examiner, University of Delhi, (iii) Chairman, Nuclear Medicine Committee (1968–78), (iv) Member, Radiopharmaceuticals Committee (1968–79), (v) Consultant, Indian Council of Medical Research, (vi) Consultant, Department of Science and Technology, Government of India, and (vii) President, Environmental Mutagen Society of India (1975–78). Internationally, he was (i) Representative of India, United Nations Scientific Committee on the Effects of Atomic Radiation (UNSCEAR), (ii) Member, Committee IV, International Commission on Radiological Protection (ICRP) (1973–77), (iii) Member, International Commission for Protection against Environmental Mutagens and Carcinogens (ICPEMC), and (iv) Member, Editorial Board, International Journal of Nuclear Medicine and Biology.

In the first meeting of the AMPI held in July 1976 in the Modular Laboratory, BARC, Dr. Sundaram was unanimously elected as President of the AMPI for the two-year term covering 1976–78. Soon thereafter, Dr. Sundaram and I, as treasurer of the AMPI, went to the Registrar's Office in Worli, Mumbai, for the formal registration of the Association as a society with the Maharashtra Government. The AMPI had several Executive Committee meetings in Dr. Sundarams's office during these formative years. Dr. Sundaram's advice, suggestions, and continuous interactions with the committee members were very valuable. It is probably in the fitness of things that Dr. Sundaram was the first member to be formally enrolled as an AMPI Life Member; his membership number was LM 001.

Dr. Sundaram is survived by his wife, Mrs. Parvathy, daughter, Mrs. Chitra, son-in-law, Mr. Srinivasan, son, Mr. Ravi, daughter-in-law, Mrs. Hema, and his grand children.

(I sincerely thank Dr. K. B. Sainis, Director, Bio-Medical Group, BARC, for providing valuable information for incorporation into this obituary note.)

